# Gliome du nerf optique révélé par un strabisme divergent

**DOI:** 10.11604/pamj.2014.17.256.3364

**Published:** 2014-04-08

**Authors:** Hanan Handor, Mina Laghmari, Zouheir Hafidi, Rajae Daoudi

**Affiliations:** 1 Université Mohammed V Souissi, service d'ophtalmologie A de l'hôpital des spécialités, Centre hospitalier universitaire, Rabat, Maroc

**Keywords:** Gliome, nerf optique, strabisme, glioma, optic nerve, strabismus

## Abstract

Les gliomes des nerfs optiques sont des tumeurs rares qui s'observent essentiellement chez l'enfant. L'exophtalmie et le strabisme sont les principaux signes révélateurs de la maladie. La neuroimagerie et notamment l'imagerie par résonnance magnétique est d'un grand apport dans le diagnostic et le suivi de ces tumeurs. La prise en charge thérapeutique de ces gliomes fait appel à différents moyens: l'exérèse chirurgicale, la chimiothérapie, la radiothérapie ou l'abstention sous surveillance. Les indications doivent être discutées au cas par cas.

## Introduction

Les gliomes des nerfs optiques sont des tumeurs rares qui s'observent essentiellement chez l'enfant. L'exophtalmie et le strabisme sont les principaux signes révélateurs de la maladie. Nous rapportons à ce juste titre un cas de gliome des nerfs optiques révélé par un strabisme divergent.

## Patient et observation

**Figure 1:**
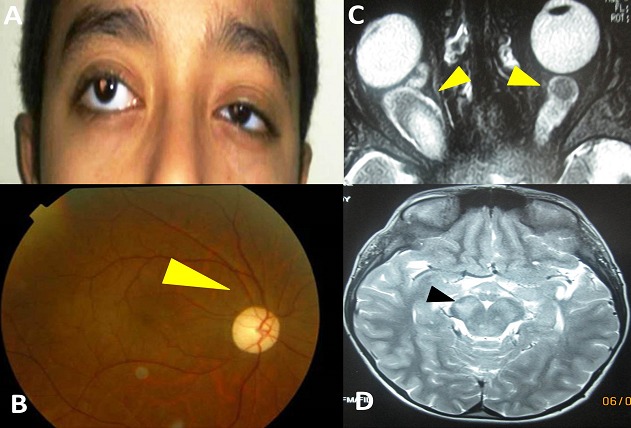
A: strabisme divergent et exophtalmie droite; B) atrophie optique au fond d’œil (flèche); C) IRM en coupe axiale objectivant une hypertrophie des deux nerfs optiques (flèches); D) IRM en coupe axiale objectivant des anomalies de signal au niveau du mésencéphale (flèche)

Il s′agit d′un garçon âgé de 8 ans, né de parents consanguins, suivi depuis l′âge de 2 ans pour un strabisme divergent précoce. L′enfant est traité par correction optique totale et rééducation de l′amblyopie jusqu′à l′âge de 6 ans. Devant la non amélioration de son état il nous a été adressé pour prise en charge. L′examen ophtalmologique a noté un strabisme divergent à grand angle constant sur l′oeil droit avec une exophtalmie modérée (Figure 1A). L'acuité visuelle corrigée était réduiteà compte les doigts de près à droite et à 3/10 àgauche. L'examen du fond d'oeil a mis en évidence une atrophie optique droite (Figure 1B). L'examen somatique a retrouvé des taches café au lait au niveau du dos. Une IRM a été demandée révélant un aspect de gliome des deux nerfs optiques (Figure 1C) associés à de nombreuses lésions infra et supratentorielles très évocatrices de foyers gliomateux cérébraux (Figure 1D). L′enfant a été adressé en neurochirurgie pour prise en charge. L′abstention thérapeutique a été préconisée avec surveillance régulière.

## Discussion

Les gliomes du nerf optique sont des tumeurs rares qui s'observent essentiellement chez l'enfant [1]. L'atteinte bilatérale des nerfs optiques est considérée comme une caractéristique de ces gliomes dans le cadre de la neurofribromatose de type I (NF1) [2] comme c'est le cas de notre patient. L'exophtalmie et le strabisme sont souvent révélateurs de la maladie. L'imagerie par résonnance magnétique est un examen clé dans l'exploration de ces gliomes. Elle permet non seulement d’étudier les nerfs optiques mais aussi de rechercher une éventuelle extension de ces gliomes à l'orbite, au chiasma et aux structures intracrâniennes particulièrement en cas d'association à une NF1 [3]. Elle a aussi l'avantage d’être mois irradiante comparée à la tomodensitométrie. La prise en charge thérapeutique va de la simple surveillance clinique et radiologique, à un traitement chirurgical ou une chimiothérapie ou radiothérapie. Les indications thérapeutiques sont discutées au cas par cas et ceci en fonction de l'acuité visuelle, du degré d'exophtalmie et de l'extension de la tumeur [4]. En cas de NF1 la radiothérapie est formellement contre indiquée du fait des complications qu'elle peut entraîner [5]. Dans notre cas, l'abstention thérapeutique avec surveillance clinique et radiologique a été préconisée. Ces contrôles se font tous les six mois, nous n'avons pas noté à ce jour de progression des lésions. Selon Tow et al, le pronostic des gliomes des voies optiques associés à une NF1 semblentêtre meilleur [6].

## Conclusion

A travers cette observation nous soulignons l'importance de réaliser régulièrement un examen du fond d'oeil devant tout strabisme précoce notamment divergent. Le moindre doute sur une atteinte organique doit faire réaliser une neuro imagerie afin de ne pas méconnaitre une pathologie qui évolue à bas bruit et qui risque de compromettre non seulement le pronostic fonctionnel mais aussi le pronostic vital.
